# Perspective of Future SERS Clinical Application Based on Current Status of Raman Spectroscopy Clinical Trials

**DOI:** 10.3389/fchem.2021.665841

**Published:** 2021-07-20

**Authors:** Xi Xi, Chongyang Liang

**Affiliations:** ^1^ Department of Biopharmacy, School of Pharmaceutical Sciences, Jilin University, Changchun, China; ^2^ School of pharmaceutical science, Institute of Frontier Medical Science, Jilin University, Changchun, China

**Keywords:** surface-enhanced raman spectroscopy, clinical trial, raman spectroscopy, disease, sample selection

## Abstract

Raman spectroscopy has emerged as a promising tool in biomedical analysis and clinical diagnosis. The development of surface-enhanced Raman scattering spectroscopy (SERS) improved the detection limit with ultrahigh sensitivity and simplicity. More and more Raman spectroscopy clinical trials (R-PCT) have been conducted recently. However, there is a lack of an up-to-date review summarizing the current status of Raman clinical trials performed until now. Hence, the clinical trials for Raman were retrieved from the International Clinical Trials Registration Platform. We summarized the clinical characteristics of 55 registered Raman spectroscopy clinical trials (R-RSCTs) and 44 published Raman spectroscopy clinical trials (P-RSCTs). This review could assist researchers and clinicians to understand the current status of Raman spectroscopy clinical research and perhaps could benefit the reasonable and accurate design of future SERS studies.

## Introduction

After the discovery of inelastic scattering of light by CV Raman in 1928, the phenomenon was reported in biomedical application for the first time in 1970 (Raman and Krishnan, 1928; Lord and Yu, 1970). In the progress of light scattering, most photons maintain the same energy and wavelength after interacting with matter (Raman and Krishnan, 1928; Auner et al., 2018). But a very small portion of photons is linearly inelastically scattered, resulting in loss of energy, and a longer wavelength (Raman and Krishnan, 1928; Wang et al., 2018). The shift of the wavelength is called Raman shift, which is inversely proportional to the change in the photons’ wavelength (Jones et al., 2019). The intensity of the Raman spectrum against Raman shift was expressed in wavenumbers with the units of cm^−1^ (Raman and Krishnan, 1928; Pilot et al., 2019). The Raman spectrum gives a directly objective picture of the molecular composition (Sahu et al., 2013).

The measurement of Raman spectroscopy is a fast, label-free, and noninvasive progress. Hence, it has many advantages in biomedical applications. The harmfulness of laser used in Raman spectroscopy can be reduced by selecting the right wavelength and power, and Raman signals provide the molecular information of tissue and cells directly (Lee et al., 2019; Chaiken and Peterson, 2021). But the natural intensity of the Raman signal was low, which resulted in low signal-to-noise ratios (Pence and Mahadevan-Jansen, 2016). In recent years, different types of Raman spectroscopy were developed to improve the sensitivity and specificity of Raman scattering, including resonance Raman spectroscopy (RRS), surface-enhanced Raman spectroscopy (SERS), and tip-enhanced Raman spectroscopy (TERS) (Bailo and Deckert, 2008; Stiles et al., 2008; Robert, 2009). The enhancement of Raman signal for RRS, SERS, and TERS was reported by a factor of 10^2^ to 10^6^, 10^5^ to 10^10^, and 10^10^, respectively (Auner et al., 2018). The enhancement of Raman spectroscopy attracted attention in clinics because of its high sensitivity (Cialla-May et al., 2017).

SERS is particularly interesting because the Raman signal can be controlled by modifying a designated probe on the surface to detect the specific analytes (Stiles et al., 2008). Raman signals of molecules adsorbed on the metal surface were amplified by generating a localized surface plasmon resonance under an incident electromagnetic field (Lee and Tseng, 2018; Pilot et al., 2019). Based on the knowledge of molecular interaction *in vivo,* such as the antibody–antigen and complementary sequences of DNA and RNA, SERS is employed to quantify drugs and biomolecules in complex systems such as blood and tissue due to its high sensitivity and specificity (Henry et al., 2016; Xue et al., 2018). SERS was also developed to be applied in surgical margin guidance such as ovarian cancer and brain tumor (Jiang et al., 2019). SERS preferred liquid samples, for example, biofluids and cells, due to the additive metallic nanoparticles and the limited detection distance (less than tens of nanometers) between analytes and metallic surfaces (Pence and Mahadevan-Jansen, 2016). To develop SERS and Raman spectroscopy better, it is necessary to understand the current status of their clinical trials.

Raman spectroscopy has been in clinical phases since 2003 (U.S. National Library of Medicine, 2003). Until now, there has been no review to characterize the clinical status of Raman spectroscopy clinical trials. In this review, we retrieved the registered Raman spectroscopy clinical trials (R-RSCTs) in trial registries of ICTRP with standardized process requirements for the first time. We summarized basic clinical characteristics, disease classification, and sample classification in ongoing and completed clinical trials to update the current status. Published Raman spectroscopy clinical trials (P-PSCTs) were also retrieved and summarized due to the lack of updated results of the R-RSCTs. Moreover, the current SERS clinical application was summarized, and its future prospect was discussed based on the above results. This could benefit the reasonable and accurate designs of future SERS studies.

## The Current Status of Raman Spectroscopy Clinical Trials: The Methods

### Search Strategy

**FIGURE 1:**
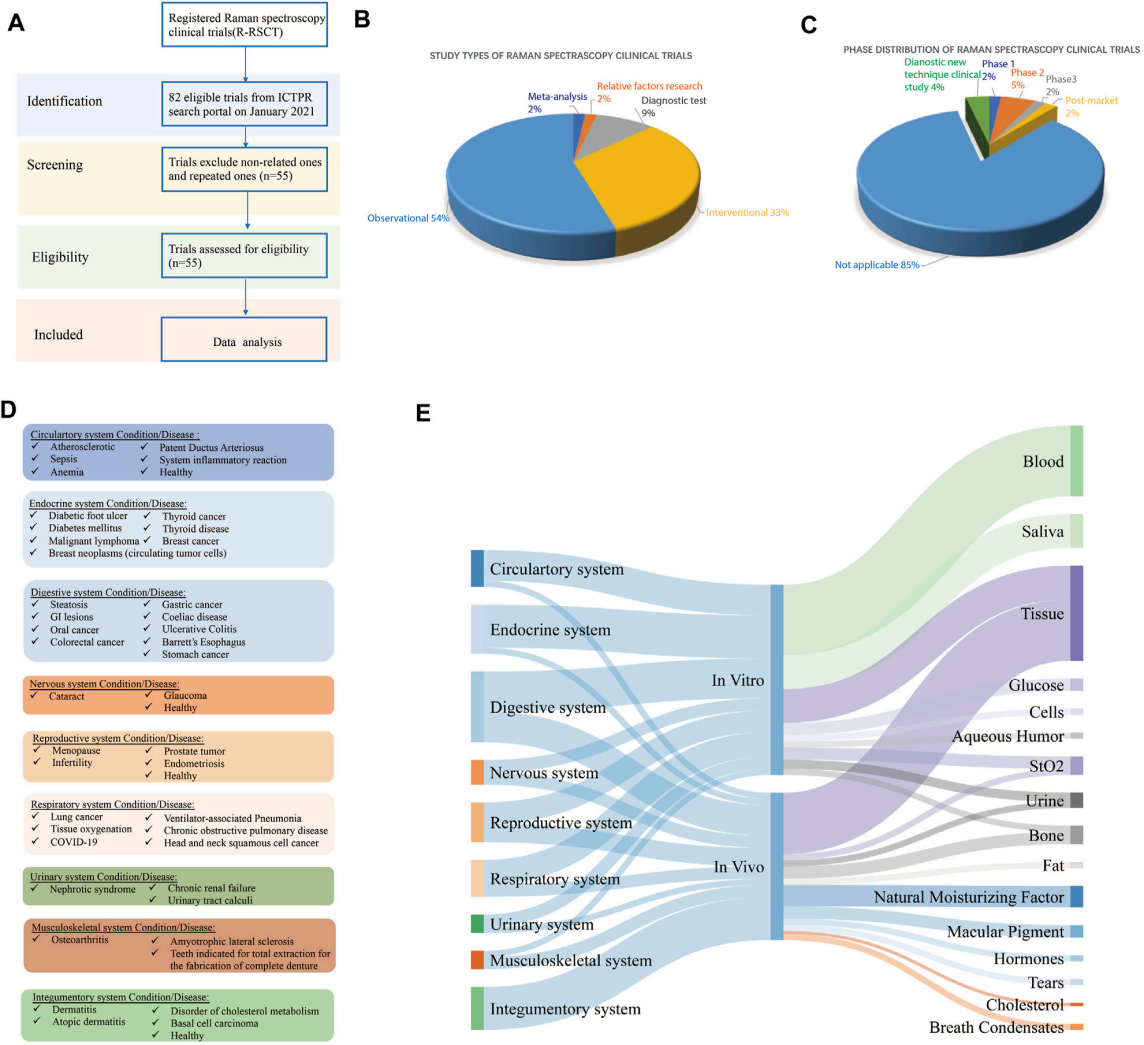
**(A)** Flowchart of Raman spectroscopy clinical trial selection, **(B)** characterization of study types of registered Raman spectroscopy clinical trials (R-RSCTs), **(C)** phase distribution of R-RSCTs, **(D)** disease classification of R-RSCTs according to physiological systems, and **(E)** the Sankey diagram of physiological systems, *in vivo/in vitro*, and sample types in R-RSCTs.

The preparation for the data of registered Raman spectroscopy clinical trials (R-RSCTs) was conducted (Figure 1): the registration database of WHO registries through the International Clinical Trials Registry Platform (ICTRP) Search Portal (http://apps.who.int/trialsearch) was used to get registration items concerning R-RSCTs by searching for the key word “Raman.” The records of non-related ones will be excluded one by one.

The preparation for the data of published Raman spectroscopy clinical trials (P-RSCTs) was conducted: the search term used was “Raman” in PubMed with the article type restriction as “Clinical trials.” The clinical studies were retrieved and followed PRISMA guidelines.

### Data Selection and Extraction

#### Trials Selection and Data Extraction

The retrieved records from the database of WHO registries were exported to Microsoft Excel. The titles, abstracts, and full texts were screened by the first author (XX) to exclude the non-related Raman spectroscopy records. The following data of Raman spectroscopy clinical trials were extracted: a combination of title, abstract, and full-text screen was performed by the first author (XX) to exclude any record that was not a Raman trial or repeated ones. The following data for trial records were extracted: trial identifier, study title, date of registration, register source, trial phase, recruitment status, anticipated enrolment sample size, condition studied, study type, sample type, *in vitro/vivo*, and the number of evaluation factors (Fan et al., 2020). However, the information of R-RSCTs in ICTRP was not updated on time, which did not reflect the present research status of completed RSCTs (Al-Durra et al., 2020).

The retrieved articles from PubMed were thoroughly screened by the first author (XX), and the following information of published Raman spectroscopy clinical trials was extracted: the Raman type, date of publication, sample type, sample size, condition studied, *in vitro/vivo*, the number of evaluation factors, sensitivity, and specificity.

#### Discrepancies

Data extraction was performed by two authors (XX and CL). Any discrepancies were resolved between the two authors.

### Data Analysis

Descriptive statistics were used to characterize the trials extracted from the ICTRP Search Portal. All statistical analyses were performed using Microsoft Excel. Missing values were excluded from the analysis unless indicated.

### Data Visualization

The flowchart was drawn with Adobe Illustrator. The pie chart was drawn with Microsoft Excel 8.0 (Microsoft, Radmond, United States). Sankey diagrams were generated with SankeyMATIC for data visualization. The illustration figure was drawn with BioRender.

## The Current Status of Raman Spectroscopy Clinical Trials: The Results

### Characterization of Registered Raman Spectroscopy Clinical Trials

We retrieved 82 records from the ICTRP database, of which 25 records were excluded because their interventions were not Raman spectroscopy (Figure 1A). Two records were excluded after full-text screening since they were the repeated ones. A total of 55 records in the ICTRP were analyzed for basic trial characteristics (Figure 1A). In Table 1, we found that 55 trials were registered in the following seven registries: Australian New Zealand Clinical Trials Registry (ANZCTR) (1), ClinicalTrials.gov (24), Chinese Clinical Trials Registry (ChiCTR) (8), Clinical Trials Registry-India (CTRI) (4), International Standard Randomized Controlled Trial Number (ISRCTN) (2), Japan Primary Registries Network (JRPN) (11), and German Clinical Trials Register (5). For the recruitment status of these trials, 20 (36.36%) are actively recruiting subjects, and most (64%) of the remaining trials have not yet started to recruit participants. As shown in Figure 1B, the study type of 30 (54.5%) R-RSCTs was for “Observational,” followed by “Interventional” (18, 32.7%), “Diagnostic test” (5, 9.1%), “relevant factors research” (1, 1.8%), and “meta-analysis” (1, 1.8%). An “Observational study” aims to observe patients or measure certain outcomes without any specific intervention, and patients will not be assigned into different groups. An “Interventional study” aims to evaluate one or more particular interventions on participants, and participants will be created into different groups. The “Diagnostic test” is a study design to evaluate diagnostic accuracy. “Relative factor research” is a study to investigate multiple factors during disease diagnosis, prognosis, and treatment efficacy evaluations. “Meta-analysis” is a statistical process that combines the findings from individual studies.

**TABLE 1:** Characteristics of registered Raman spectroscopy clinical trials.

Characteristic	Category	Number	Percentage
Source register	ANZCTR	1	1.82
	Clinicaltrials.gov	24	43.64
	ChiCTR	8	14.55
	CTRI	4	7.27
	ISRCTN	2	3.64
	JPRN	11	20.00
	German clinical trial register	5	9.09
Recruitment status	Not recruiting	35	63.64
	Recruiting	20	36.36
Target size	≦50	27	49.09
	51–100	10	18.18
	101–200	9	16.36
	201–500	4	7.27
	501–1,000	3	5.45
	1,001–10,000	2	3.64
*In vitro/in vivo*	*In vitro*	31	56.36
	*In vivo*	24	43.64
Evaluation factors	Single	8	14.55
	Multiple (image and fingerprint)	47	85.45
Study type	Observational	30	54.55
	Diagnostic test	5	9.09
	Interventional	18	32.73
	Relative factors research	1	1.82
	Meta-analysis	1	1.82
Phase	Phase 1	1	1.82
	Phase 2	3	5.45
	Phase 3	1	1.82
	Post-market	1	1.82
	Not applicable	47	85.45
	Diagnostic new technique clinical study	2	3.64

For the phase distribution, most of the trials were under the phase “Not Applicable” in Figure 1C. The main reason may be the failure of recruitment. One trial (No. CTRI/2018/01/011139) was under the post-market phase to analyze the physicochemical interactions of the dentin–resin interface (Girija, 2018). One trial (No. NCT00060580) was under Phase 1 to measure the amount of the pigment lutein in the retina (U.S. National Library of Medicine, 2003). Three trials (No. NCT02033512, No. NCT02621853, and No. CTRI/2009/091/000851) were under Phase 2 to detect hormones, fat, and saliva, respectively (U.S. National Library of Medicine, 2014; U.S. National Library of Medicine, 2015; Vedang, 2009). One trial (No. ChiCTR1800016644) was in Phase 3 to monitor dermatitis degree in treatment of asthma by acupoint sticking therapy (Liu, 2018). Two trials (No. ChiCTR-RDC-17012611 and No. ChiCTR1800015711) were categorized to diagnostic new technique clinical study (Qiu, 2017; Qian, 2018). The sample size is mostly distributed in the middle and small sample size, and 83.6% is less than 200 subjects. Of these R-RSCTs, nearly 85% of evaluation factors were multiple. Only eight trials were evaluated by the single factor. In total, 31 trials were conducted *in vitro* and 24 trials *in vivo*. These characteristics above and others of the R-RSCTs are summarized in Table 1.

In Figure 1D, 47 diseases of R-RSCTs were classified into nine types according to the physiological systems. The digestive system (nine diseases) had the most number of diseases, followed by the endocrine system (seven diseases), circulatory system (six diseases), and respiratory system (six diseases). It should be noticed that no diseases from the immune system were chosen as the condition in R-RSCTs. In Figure 1E, the samples corresponded to *in vitro/in vivo*, and the physiological systems were shown as the Sankey diagram. The clinical trials conducted *in vitro* have nine types of samples. Blood, saliva, and tissue were taken in most of the trials.

### Characterization of Published Raman Spectroscopy Clinical Trials

The majority of the above registrars still need to be completed, and the completed clinical trials did not update their results in real time. Therefore, we collected 44 results of the published Raman spectroscopy clinical trials in PubMed to obtain the current status. There are ten types of Raman spectra which have been studied in the clinic, as shown in Table 2: “Confocal Raman” (11), “Probe Raman” (10), conventional Raman spectrometer (6), “Raman microscope” (4), “Resonance Raman” (4), “Micro-Raman” (3), “Transcutaneous Raman” (2), SERS (2), “FT-Raman” (1), and “Kerr-gated Raman” (1). 22 conditions including healthy and 21 diseases are summarized in Table 2. The published articles showed that Raman spectroscopy was widely used *in vivo* and *in vitro*. “Confocal Raman,” “Resonance Raman,” and “Transcutaneous Raman” were only conducted *in vivo*. Transcutaneous Raman and Confocal Raman were both approved to meet the clinical accuracy requirement in the noninvasive detection of glucose *in vivo*. Confocal Raman, Probe Raman, and conventional Raman were applied to evaluate skin and components of skin under healthy and dermatitis conditions. Probe Raman, Raman microscope, conventional Raman, Kerr-gated Raman, and SERS were used in the diagnosis of 11 types of cancer. FT-Raman and Transcutaneous Raman could provide the fingerprints of bone *in vivo* and teeth *in vitro*. The sample size of most studies was less than 100, which accounted for 90.9% of P-RSCTs. P-RSCTs were all single-site studies, and no multicenter clinical trials were published. Multicenter studies were widely recognized to eliminate the bias in a single-site study and generate more convincible evidence by large numbers of hospitals and patients (Zheng and Zelen, 2008). Hence, the published results indicated the primary evaluation of Raman spectroscopy in the clinic.

**TABLE 2:** Characteristics of published Raman spectroscopy clinical trials.

Raman type	Number	Sample type	*In vivo/vitro*	Condition/disease	References
Confocal Raman	11	Ibuprofen	*In vivo*	Healthy	
		Skin		Atopic dermatitis	Chrit et al. (2005); Chrit et al. (2006); Richters et al. (2017)
				Healthy	
		Dermal water		Dermatitis	Nakagawa et al. (2010)
		Retinyl acetate		Healthy	Lee et al. (2015); Dos Santos et al. (2017)
		Hormone		Climacteric symptom	Botelho et al. (2014)
		Oil		Healthy	Choe et al. (2015)
		Glucose		Diabetes	Chaiken et al. (2005)
		*Trans*-urocanic acid		Healthy	Egawa and Iwaki, (2008)
Probe Raman	10	Tissue	*In vitro*	Brain tumor	Koljenovic et al. (2005)
			*In vivo*	Soft tissue sarcomas	Kanter et al. (2009); Huang et al. (2010); Bergholt et al. (2016); Nguyen et al. (2016); Lin et al. (2017)
				Nasopharyngeal carcinoma	
				Gastric cancer	
				Colorectal cancer	
				Cervical dysplasia	
				Dermatitis	
		Skin		Skin cancer	Schleusener et al. (2015)
		Filaggrin		Atopic dermatitis	González et al. (2011)
Raman microscope	4	Serum	*In vitro*	Oral caner	Sahu et al. (2013)
		Hydroxyethyl starch	*Ex vivo*	Renal graft	Vuiblet et al. (2016)
		Salivary gland	*In vivo*	Sjogren’s syndrome	Xue et al. (2014)
		Beta-tricalcium phosphate		Osseointegration	Pascaretti-Grizon et al. (2017)
				Sinus lift	
				Bone graft	
Micro-Raman	3	Luting agents	*In vitro*	Healthy	Lohbauer et al. (2010); Navarra et al. (2012)
				Natural cavities in teeth	
		Tissue	*In vivo*	Periodontal inflammation	Camerlingo et al. (2014)
Resonance Raman	4	Carotenoids	*In vivo*	Healthy	Hesterberg et al. (2009)
		Macular pigment			Tanito et al. (2012)
		Lycopene and ß-carotene			Blume-Peytavi et al. (2009)
		Human molar		Healthy/aging	Ager et al. (2006)
Transcutaneous Raman	2	Glucose	*In vivo*	Healthy	Enejder et al. (2005)
		Bone			Matousek et al. (2006)
Raman spectrometer	6	Serum	*In vitro*	Healthy	Rohleder et al. (2005)
				Diabetes	
		Tissue		Ovarian cancer	Crow et al. (2003); Maheedhar et al. (2008)
				Prostate cancer	
			*In vivo*	Skin cancer	Sigurdsson et al. (2004); Moncada et al. (2016)
				Melasma	
		Urea		Healthy	Egawa and Sato, (2015)
FT-Raman	1	Tooth	*In vitro*	Healthy	Paula et al. (2010)
Kerr-gated Raman	1	Tissue	*In vitro*	Prostate cancer	Prieto et al. (2005)
				Bladder cancer	
SERS	2	Tissue	*In vitro*	Colon cancer	Zavaleta et al. (2013)
		Serum		Oral squamous cell carcinoma	Tan et al. (2017)

**FIGURE 2:**
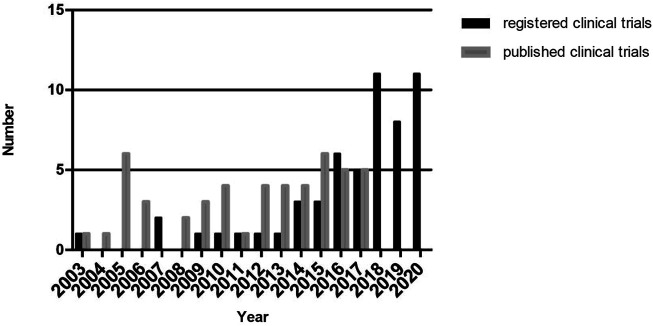
Number of registered and published Raman spectroscopy clinical trials from 2003 to 2020.

We summarized the present situation of 55 R-RSCTs and 44 P-RSCTs in this review. Only three studies were published before 2008. From 2003 to 2017, both R-RCTS and P-RCTs gradually increased. From 2018 to 2020, R-RCTs raised up rapidly to around 10 clinical trials each year. No published results were obtained in PubMed since 2018. Collectively, the results are shown in Figure 2. We look forward to the release of these registered clinical trials in the next 5 years.

## The Current Status of SERS Clinical Trials: The Results

**FIGURE 3:**
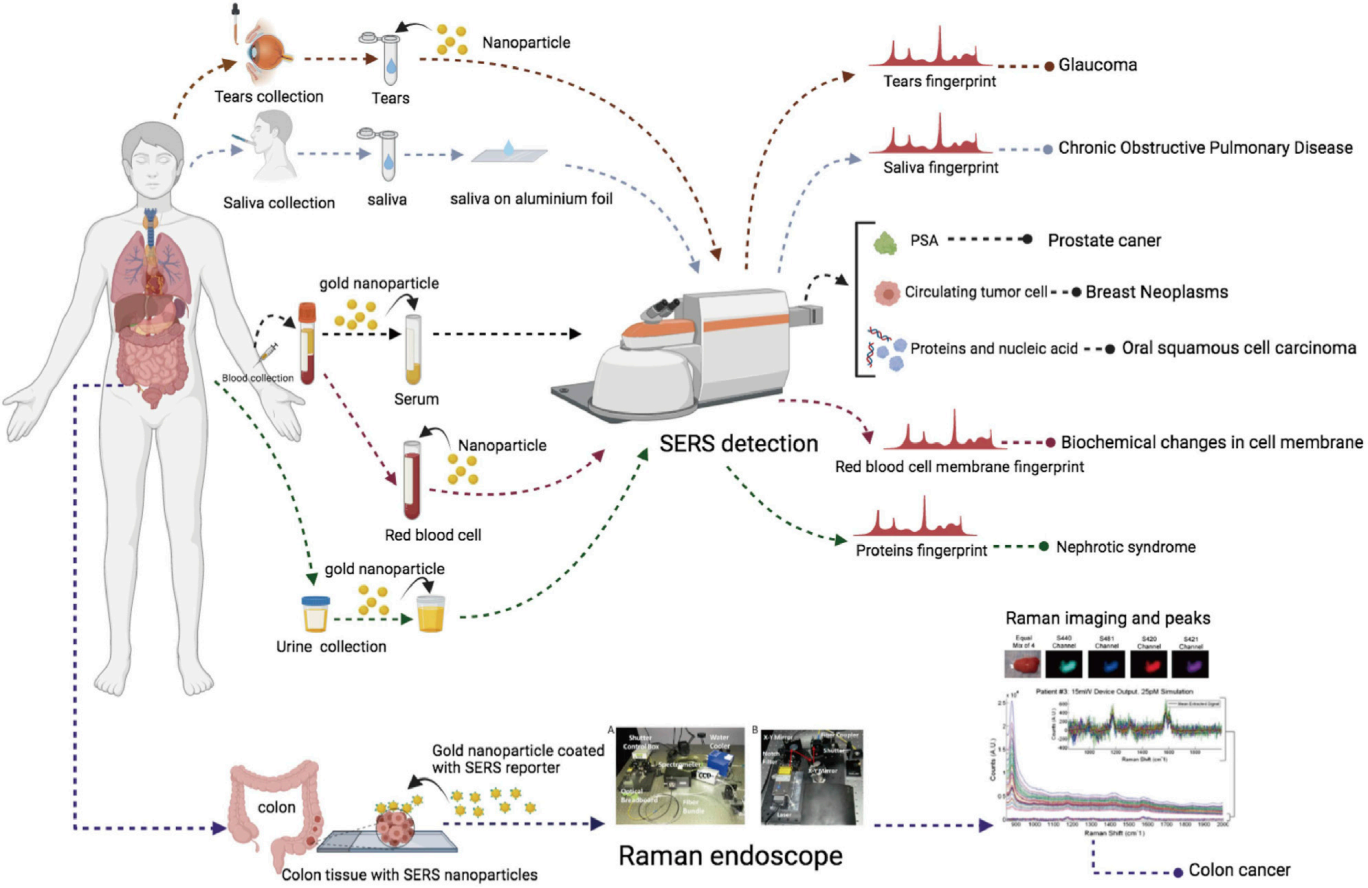
Illustration of SERS registered and published clinical trials.

There are six registered clinical trials (Table 3) related to SERS and two published clinical trials. The illustration of samples and collected outcomes from these trials are shown in Figure 3. The study type of five registered trials was “Observational” and one trial was “Diagnostic test.” The interventional/observational models were “Factorial,” “Cohort,” and “Case–control.” A “Factorial” study aims to evaluate two interventions in the same trial and their interactions between two interventions. A “Cohort” study aims to measure the effect of a suspected risk factor in both groups of people who have a certain condition and who have no condition. A “Case–control” study aims to determine factors associated with a certain condition. Two trials were registered in 2019 and four trials in 2020. Only two trials are actively recruiting subjects. The sample size of four trials was distributed in middle and small sizes. All the trials were under the phase “Not applicable.” The sample types were blood, urine, saliva, and tears. All the trials were conducted *in vitro*. According to the published results of two studies, SERS had a sensitivity of 80.7% and a specificity of 84.1% in the diagnosis of oral squamous cell carcinoma by analyzing the fingerprints of blood and sensitivity of 326-fM SERS nanoparticles in colon tumor tissue (Zavaleta et al., 2013; Tan et al., 2017). According to the trials, it indicated that SERS preferred the sample type of biofluids and depended on metal nanoparticles to magnify signals.

**TABLE 3:** Characteristic of registered SERS clinical trials.

Register number	Title	Date registration	Condition/disease	Sample type	Study type	Rec**r**uitment status	Target size	*In vivo/in vitro*	Phase	Interventional/obse**r**vational model	References
ChiCTR2000037082	Construction of artificial intelligence–assisted prostate tumor early diagnosis system based on surface-enhanced Raman spectroscopy	August 26, 2020	Prostate tumor	Blood	Diagnostic test	Recruiting	2000	*In vitro*	N/A	Factorial	Wang, (2020)
NCT04239105	Detection and analysis of circulating tumor cells (CTCs) in patients with breast cancer using a novel microfluidic and Raman spectrum device	December 31, 2019	Breast neoplasms (circulating tumor cells)	Blood	Observational	Not recruiting	120	*In vitro*	N/A	Cohort	U.S. National Library of Medicine. (2020a)
NCT04311684	Development and validation of hybrid Brillouin–Raman spectroscopy for noninvasive assessment of mechanochemical properties of urine proteins as biomarkers of kidney diseases	March 14, 2020	Nephrotic syndrome	Urine protein	Observational	Recruiting	80	*In vitro*	N/A	Cohort	U.S. National Library of Medicine. (2020b)
NCT04628962	Raman analysis of saliva as a biomarker of COPD	November 09, 2020	Chronic obstructive pulmonary disease	Saliva	Observational	Recruiting	250	*In vitro*	N/A	Case–control	U.S. National Library of Medicine. (2020c)
CTRI/2019/06/019890	Human tear sample studies using high-performance liquid chromatography with laser-induced fluorescence (HPLC-LIF) and surface-enhanced Raman spectroscopy (SERS) techniques	27–06-2019	Glaucoma	Tears	Observational	Not recruiting	35	*In vitro*	N/A	N/A	Adigal et al. (2019)
CTRI/2020/07/026418	Design and development of an optical setup for cell membrane-targeted surface-enhanced Raman spectroscopy using vortex beams	07–07-2020	Healthy	Blood	Observational	Not recruiting	30	*In vitro*	N/A	N/A	Ghana Shyam, (2020)

## Discussion and Perspective of Future SERS Clinical Application

This review provides an overview of the current status of Raman spectroscopy clinical registration information and published articles. It is a new and extensive survey of R-RSCTs and P-RSCTs. Most R-RSCTs are registered on platforms from the United States, Japan, China, and Germany. Compared with preclinical studies, the ongoing R-RSCTs are very rare, and most researchers focused on developing new technology in cellular and animal models. As we mentioned above, the completed R-RSCTs in the WHO platform did not update their results in real time. Moreover, the clinical protocol of some studies in the publication was different from the registered one. It is difficult to analyze the results of R-RSCTs. Many researchers only registered a trial about Raman spectroscopy without recruiting any participants. In the summary of P-RSCTs, we found that P-RSCTs were subjected to a single-center study with a sample size of fewer than 100 subjects. Although Raman spectroscopy had many advantages, some types of Raman spectroscopy failed to continue to be developed in the clinic such as the instrument of Kerr-gated Raman, which was filled in two rooms (Prieto et al., 2005). Hence, we suggest that the researchers from universities and research centers may collaborate more with clinicians and industry sponsors to conduct large-scale, high-quality, and multicenter R-RSCTs and publish their results in the corresponding registration platforms. SERS also has the same problems above, and it is in the early stage of clinical development. Most clinicians are familiar with the technologies such as computed tomography (CT) and nuclear magnetic resonance (NMR), but unfamiliar with SERS, and other Raman spectra. This resulted in the slow development of R-RSCTs and P-RSCTs.

SERS depends on the metal surface but is also limited by the interaction surface in the clinic. Most clinical trials chose the wavelength of laser at 785 nm to excite the light scattering and collect the Raman signals, which proved efficiency and safety of the laser (Sigurdsson et al., 2004). But the penetration distance *in vivo* of the laser was limited to less than 10 mm (Nakagawa et al., 2010; Egawa and Sato, 2015). Hence, SERS can only be used *in vitro* and *ex vivo* to make metallic surfaces and samples close enough. There are two methods to use SERS in biomedical research. One is to add nanoparticles without SERS reporter in the samples and amplify the intrinsic Raman signals. Another one is to administrate the nanoparticles with SERS reporter directly to detect the reporter signals. Fingerprints require complex interpretation and the concern of reproducibility. The fluorescent background of samples also brings a low signal-to-noise ratio (SNR). The second method may be a better choice in clinical application due to its higher specificity and higher SNR.

Importantly, several critical issues need to be concerned with the following. First, there is the selection of reliable biomarkers in biofluids: oncology is still the “Gold standard” of diagnosis. Applying biomarkers in diagnosis calls for researchers from basic medicine, optical spectroscopy, and clinicians from hospitals to collaborate. Second, there are multicenter and large-scale clinical trials: large-scale trials should be conducted under standard protocols to prove the advantages of SERS in the clinic compared to other techniques. Third, there is the artificial intelligence of SERS spectra: although SERS signals may have several sharp peaks, it is still difficult to analyze the intensity and the shifts of wavelength directly. Diagnosis models of diseases are necessary to be built by artificial intelligence to transform SERS spectra to a readable clinical standard immediately. Three AI-related Raman spectroscopy clinical trials were registered in 2021. Overall, we look forward to breakthrough developments of SERS in the clinic in the next 5 years.
